# Tamoxifen evolution

**DOI:** 10.1038/s41416-023-02158-5

**Published:** 2023-02-10

**Authors:** A. Howell, S. J. Howell

**Affiliations:** 1 Division of Cancer Sciences, Faculty of Biology, Medicine and Health, University of Manchester, Manchester Academic Health Science Centre, Manchester, UK; 2 Prevent Breast Cancer Centre, Wythenshawe Hospital Manchester Universities Foundation Trust, Wythenshawe, Manchester, UK; 3 Manchester Breast Centre, The Christie NHS Foundation Trust, Wilmslow Road, Manchester, UK

**Keywords:** Breast cancer, Targeted therapies

## Introduction

Tamoxifen has changed the landscape of breast cancer treatment and prevention since its introduction in the late 1960’s. We previously reviewed tamoxifen development until 2002 and here, we update the review for the 70th-anniversary edition of the British Journal of Cancer [1]. It is of interest that the first clinical use of tamoxifen was reported in the ‘Journal’ in 1971 [2].

Since there is a very large tamoxifen literature, we have chosen to cite the first critical initial studies in the treatment of advanced breast cancer, adjuvant therapy and it’s use for prevention. We follow this early development by overviews of subsequent trials which establish the current place of tamoxifen in treatment in each area. For advanced disease, reviews summarise the place of tamoxifen amongst other treatments for advanced disease [3, 4]. For adjuvant trials, the overviews produced by the Early Breast Cancer Trials Collaborative Group (EBCTCG) from Oxford are highly important since they collate individual patient data from all available randomised trials in a particular area of treatment. Reviews for prevention summarised all prevention trials using tamoxifen [5]. Tamoxifen became the treatment of first choice for virtually all clinical situations but the advent of well-tolerated third-generation aromatase inhibitors and selective oestrogen receptor degraders (SERDs) has resulted in a decline in the use of tamoxifen in most settings. This decline may be exacerbated if the development of oral selective oestrogen receptor down-regulators (SERDs) is successful, although tamoxifen remains on the WHO’s list of essential medicines [6, 7].

## Origins of tamoxifen

The first endocrine therapy was surgical oophorectomy in young women with advanced disease [8]. This was followed by the first-ever randomised oncology trial which examined the role of adjuvant ovarian irradiation and started recruitment in 1948 [9]. The discovery of ovarian oestrogens by Allen and Doisy [10] initiated a successful search for and development of the long-acting synthetic oestrogens, stilboestrol [11] and the triphenylethylenes [12]. Stiboestrol and two analogues of triphenylethylene, (trichlorophenylethylene (Gynosome) and trimethylphenylethylene) entered clinical trials for advanced breast cancer in the early 1940s [13]. Stilboestrol became the mainstay of oestrogen therapy until the development of tamoxifen 30 years later. Trichlorophenylethylene later formed the basis of MER25 and Clomiphene, the first antiestrogens, which were clinically developed in the 1950s in two small trials in advanced breast cancer, but this development was not pursued [14]. Trimethylphenylethylene (M260) formed the basis of the development of tamoxifen in the 1960s. The lead compound for tamoxifen development was found to be a mixture of *cis* and *trans* isomers. Separation of the *cis* isomer which was found to be a pure oestrogen (ICI 47,699) from the *trans* isomer, a mixed oestrogen/anti-oestrogen, now in the clinic as tamoxifen (ICI 46,474), is likely to have enhanced tamoxifen’s success [15, 16].

## Development in advanced breast cancer

The first single-arm Phase 2 trial of tamoxifen was initiated in 1969. Forty-six patients were recruited and 22% were assessed as responding to treatment. The response rates to tamoxifen were compared with the hospital records of 64 patients treated will stilbestrol, of which 16 (25%) responded, and 60 with high-dose androgens of which 11 (18%) responded. The response rates of the compounds were, therefore, comparable but major differences in toxicity profiles were reported [2].

Tamoxifen was subsequently compared in randomised trials with other agents/surgical procedures used at the time, including stilbestrol, megestrol acetate, medroxyprogesterone acetate, fluoxymesterone, nandronolone, first and second-generation aromatase inhibitors, surgical oophorectomy and adrenalectomy. A comprehensive review of all these trials reported no significant difference in survival (24 comparisons HR = 1.02) but a higher incidence of side effects with the other therapies, including fatigue, lethargy, congestive cardiac failure, alopecia and weight gain compared with tamoxifen [3].

Randomised trials of tamoxifen versus other SERMs, synthesised in the hope of greater clinical activity, reported them to be either equally (idoxifene, toremifine) or less effective than tamoxifen (raloxifene, arzoxifene) in the treatment of advanced breast cancer [4, 17]. Thus, tamoxifen retained its place as the lead SERM and there still remains interest in developing analogues of tamoxifen with greater activity and reduced toxicity [18].

However, several companies were convinced that analogues of oestrogen were potentially more active than tamoxifen. Wakeling et al. synthesised an oestrogen analogue (ICI182,780; subsequently named fulvestrant) which was called a ‘pure’ anti-oestrogen since little agonist activity was seen in standard cell/animal assays [19]. The compound showed greater activity than tamoxifen when given preoperatively in the clinic and was also active when given to patients with advanced disease resistant to tamoxifen [20, 21]. At the previously licenced intramuscular dose of 250 mg monthly, fulvestrant was found to be equally active as tamoxifen [22]. However, when given at a dose of 500 mg, it was superior to the 250 mg dose and also to the third-generation aromatase inhibitor anastrozole in randomised trials, suggesting, indirectly, that fulvestrant may have greater activity than tamoxifen [23]. The mechanism of action of fulvestrant, in downregulating the oestrogen receptor, also offers advantages in overcoming ESR1-mediated treatment resistance to aromatase inhibition [24].

Given the difficulties of intramuscular dosing, there is great interest in oral SERDs. A promising example is elacestrant which in a recent study in second and third-line treatment showed superiority over fulvestrant at the 500 mg monthly dose, and also aromatase inhibition [25]. It seems likely that oral SERDs will replace tamoxifen once dosing and tolerability are optimised.

## Tamoxifen and early breast cancer

The relatively low toxicity of tamoxifen lead to the establishment of multiple clinical trials where the comparator with tamoxifen was the then current standard of no adjuvant therapy. The main issues to be addressed were the optimal duration of therapy and the toxicity in this early breast cancer setting. The first trial initiated in 1976 tested 1 year of tamoxifen treatment, the second 2 years and the third 5 years [26–28]. These, and multiple later adjuvant trials, were reviewed by the Early Breast Cancer Trials Collaborative Group [29]. Reductions in recurrence after about 10 years of follow-up were 21%, 29% and 47% for 1, 2 and 5 years of treatment, respectively. The corresponding mortality reductions were 12%, 17% and 26%, respectively, with a significantly significant test for trends in both recurrence and mortality.

The data from the EBCTCG led to the introduction of 5 years of tamoxifen as the standard of care as adjuvant therapy. Two trials were devised to explore the value of extending treatment to 10 years compared with 5 years [30, 31]. The ATLAS trial randomised 12,894 patients to either stop tamoxifen treatment at 5 years or to continue to complete a total of 10 years of therapy and showed a significant reduction in recurrence and mortality with the longer versus shorter treatment (recurrence at years 21.4% vs 25% and mortality 12.2% vs 15.0% for 5 and 10 years, respectively). Extended tamoxifen was associated with small increases in pulmonary embolism (HR 1.87; *P* = 0.01, and endometrial cancer (HR 1.30; *P* = 0.0002) and a reduction in ischaemic heart disease (HR 0.75; *P* = 0.02).

The ATTOM trial randomised 6,953 patients with ER-positive early breast cancer to the same approach and showed improved outcome with longer therapy with respect to breast cancer recurrence (*P* = 0.003) and breast cancer mortality (*P* = 0.05), although these data are still to be published.

These, and other, studies have led to changes in guidelines recommending extended treatment durations in women with poorer prognosis disease [32]. The quantification of the risk of late recurrence in ER + EBC and novel, predominantly clinic-genomic assays to identify those at increased risk, will hopefully guide future extended adjuvant therapy strategies [33, 34].

## Tamoxifen and aromatase inhibitors for early breast cancer

Tamoxifen showed similar treatment advantages to first and second-generation aromatase inhibitors in advanced breast cancer in the studies noted earlier [3]. Novel high potency, third-generation, aromatase inhibitors (anastrozole, letrozole and exemestane) that had significantly better targeting and thus tolerability, were tested in advanced breast cancer in the 1990s and showed, in some instances, greater activity than tamoxifen [35]. This led to the introduction of the first adjuvant trial which randomised postmenopausal women to tamoxifen or anastrozole for 5 years. Recruitment began in 2002 and demonstrated that anastrozole was significantly superior to tamoxifen for relapse-free [27] and overall survival [36].

An EBCTCG overview analysis of all available trials of 5 years of tamoxifen versus 5 years of aromatase inhibitors showed a reduction in relapse for the first 5 years and a 10-year mortality advantage for AIs (12.1% vs 14.2% RR 0.85 *P* 0.009) [6]. Tamoxifen was also compared with AIs in four trials in premenopausal women with concomitant ovarian suppression. There was a greater reduction in relapse by AIs (RR 0.79 *P* 0.0005) but, at present no significant impact on mortality [37]. Although the ATLAS and aTTom studies demonstrated improved efficacy with 10 vs 5 years of therapy, the situation with the AIs is less clear. Extending AI therapy beyond 7 or 8 years may not improve outcomes, even in higher-risk patients, with the potential for increased bone and cardiovascular toxicity with more prolonged oestrogen suppression [38]. The ‘reverse switch’ from 5 years of AI, in combination with OFS in premenopausal women, to single-agent tamoxifen for an additional 5 years is an interesting possibility that requires testing in future clinical trials.

## Tamoxifen and prevention of breast cancer

The demonstration that administration of tamoxifen at the same time as the breast cancer inducting carcinogen DMBA, reduced subsequent cancers in rats and that tamoxifen reduced contralateral breast cancer in the NATO adjuvant trial led to interest in breast cancer prevention [39, 40]. Powles at, initiated a pilot trial and later the first randomised trial of tamoxifen versus placebo in October 1986 [41]. Later randomised trials were set up in the UK & Australia/New Zealand, Italy and the USA. A meta-analysis of the trials was published in 2003 [5]. In women randomised to tamoxifen versus placebos, there was a 38% reduction in breast cancer incidence irrespective of age of the women treated. A follow-up of the UK/ANZ trial indicated a continued risk reduction up to at least 15 years after the 5-year treatment period [42]. Studies of AIs (exemestane and anastrozole) versus placebo report 50–60% reduction in breast cancer risk in postmenopausal women, which appears superior to tamoxifen. However, no head-to-head comparisons of AIs and tamoxifen have been made for cancer prevention.

## Tamoxifen dose

The first trial of tamoxifen in advanced breast cancer used daily doses of 10 mg or 20 mg [2]. The second trial used 20 mg and 40 mg and suggested a dose-response effect, although patient numbers were small [43]. An overview of adjuvant trials indicated that the standard 20 mg dose was as effective as the 40 mg dose [29]. Biomarker and pre-operative histological studies suggested little difference between 20 mg/day, 5 mg/day and 1 mg/day [44]. However, patient management cannot be planned on biomarker data alone.

Recently, DeCensi and his colleagues reported a 3-year study comparing tamoxifen 5 mg/day with placebo in women with breast intraepithelial neoplasia. At a median follow-up of 5.1 years in 500 patients, there were 14 neoplastic events with tamoxifen and 28 with placebo (HR 0.48, *P* = 0.02). There was a slight increase in hot flashes in women taking tamoxifen (*P* = 0.02). Tamoxifen 5 mg also reduced contralateral breast cancer by 75% (3 vs 12 events *P* = .02) [45]. The 5 mg dose of tamoxifen (named ‘baby-TAM’ by the investigators) was shown to be more effective in postmenopausal women, in those with low-baseline estradiol (<15.8 pg/ml) and with menopausal symptoms at baseline [46]. This is an important study and may stimulate further trials of low-dose tamoxifen to establish its position in therapy compared with the standard 20 mg dose.

There has been great interest in the use of mammographic density as a marker of responsiveness to tamoxifen [47, 48]. More recently, Eriksson et al. demonstrated that density reduction over a period of six months did not differ between doses of 20 mg, 10 mg, 5 mg and 2.5 mg of tamoxifen and that toxicity was significantly reduced at the lower doses, giving more impetus to the exploration of lower doses in future clinical trials. However, although the reliance on change in mammographic density is attractive, a recent review indicated that data supporting it as an adequate predictive or prognostic factor are not strong [49].

## Tamoxifen metabolites

Tamoxifen is metabolised to its two major active metabolites, 4-hydroxy tamoxifen and 4-hydroxy-N-desmethyl tamoxifen (endoxifen), by cytochrome P450 2D6 (CYP2D6). A patient’s intrinsic genotype of the CYP2D6 enzyme and co-administration of compounds that inhibit CYP2D6 activity have been shown to alter the serum levels of these most potent metabolites. However, the two major questions that arise are: does the intrinsic rate of metabolism and the use of CYP2D6 inhibitors (e.g., certain antidepressants) affect the clinical effectiveness of tamoxifen and if so should we use the levels of these metabolites as predictive biomarkers or even treat with the active metabolites themselves?

To try to answer the first question studies have been conducted to show that increasing the dose of the tamoxifen from 20 to 40 or even 80 mg daily can increase blood endoxifen levels [50, 51]. However, in a randomised trial of tamoxifen 40 mg vs 20 mg in women with advanced breast cancer and poor metaboliser CYP2D6 genotypes no difference in PFS rates at 6 months was seen (67.6% vs 66.7%, respectively) despite significantly higher Z-endoxifen levels in the high-dose arm (median, 89.2 nM v 51.1 nM; *P* < 0.0001) [52]. This also applied to the low-dose prevention study where CYP2D6 genotype was not related to the development of breast cancer precursor lesions in the breast [53]. In addition, a recent overview of all studies relating to the co-administration of tamoxifen and antidepressants, the major class of CYP2D6 inhibitors used in conjunction with tamoxifen, suggested that there was unlikely to be an important effects on relapse after adjuvant tamoxifen [54]. Interestingly studies using endoxifen itself for treatment are ongoing, including topical endoxifen as a method of reducing mammographic density in BC prevention [55, 56]. Unfortunately, the latter study reported that most women developed a breast rash requiring termination of treatment, despite some evidence of a reduction in mammographic density and the question remains whether we should consider 4-hydroxy-N-desmethyl-tamoxifen a novel SERM rather than truly tamoxifen itself.

## Summary

### Tamoxifen evolution

Tamoxifen’s introduction was timely as it heralded the era of powerful and, perhaps more importantly, better-tolerated agents targeting oestrogen signalling in breast cancers. Multiple randomised trials demonstrated that tamoxifen was equivalent in time to progression and survival and not inferior to the other SERMs tested. The adjuvant therapy and prevention trials versus placebo (or no treatment) indicated that 5 years of tamoxifen was the standard for the reduction of both relapse and breast cancer incidence. However, with time, improved aromatase inhibitors and the development of SERDs such as fulvestrant have led to a reduction in the indications for tamoxifen and it is now mainly used as adjuvant therapy for lower-risk premenopausal breast cancer and for prevention. A recent review of the treatment of advanced ER + ve breast cancer does not include tamoxifen at all in the suggested treatment algorithm for the disease [57].

Recent studies suggest that we may be on the brink of a new era of more widespread use of lower-dose tamoxifen, particularly for prevention in peri and postmenopausal women. Doses of about 5 mg per day are associated with fewer side effects than reported for the standard 20 mg dose. Reduction of mammographic density, endometrial proliferation, increased bone density and reduced markers of cardiovascular disease confer additional potential, and data suggest equivalence to the 20 mg dose. Further exploration of low-dose treatment is warranted, especially since more women at high risk of breast cancer are being identified in risk prediction programmes that utilise factors such as mammographic density and single-nucleotide polymorphism polygenic risk scores to introduce risk-adapted breast screening [58–60].
